# Efficient deoxygenation of waste cooking oil over Co_3_O_4_–La_2_O_3_-doped activated carbon for the production of diesel-like fuel†

**DOI:** 10.1039/c9ra09516k

**Published:** 2020-01-30

**Authors:** G. Abdulkareem-Alsultan, N. Asikin-Mijan, G. Mustafa-Alsultan, H. V. Lee, Karen Wilson, Y. H. Taufiq-Yap

**Affiliations:** Catalysis Science and Technology Research Centre (PutraCat), Faculty of Science, Universiti Putra Malaysia 43400 UPM Serdang Selangor Malaysia taufiq@upm.edu.my +603-89466758 +603-89466809; Chemical and Environmental Engineering Department, Faculty of Engineering, Universiti Putra Malaysia 43400 UPM Serdang Selangor Malaysia kreem.alsultan@yahoo.com +601-82534058; Chancellery Office, Universiti Malaysia Sabah 88400 Kota Kinabalu Sabah Malaysia; Nanotechnology & Catalysis Research Centre (NanoCat), Institute of Postgraduate Studies, University Malaya 50603 Kuala Lumpur Malaysia; School of Science, RMIT University Melbourne VIC 3001 Australia; Maintenance of Rotary Machine Equipment, South Refineries Company Basra Iraq

## Abstract

Untreated waste cooking oil (WCO) with significant levels of water and fatty acids (FFAs) was deoxygenated over Co_3_O_4_–La_2_O_3_/AC_nano_ catalysts under an inert flow of N_2_ in a micro-batch closed system for the production of green diesel. The primary reaction mechanism was found to be the decarbonylation/decarboxylation (deCOx) pathway in the Co_3_O_4_–La_2_O_3_/AC_nano_-catalyzed reaction. The effect of cobalt doping, catalyst loading, different deoxygenation (DO) systems, temperature and time were investigated. The results indicated that among the various cobalt doping levels (between 5 and 25 wt%), the maximum catalytic activity was exhibited with the Co : La ratio of 20 : 20 wt/wt% DO under N_2_ flow, which yielded 58% hydrocarbons with majority diesel-range (*n*-(C_15_ + C_17_)) selectivity (∼63%), using 3 wt% catalyst loading at a temperature of 350 °C within 180 min. Interestingly, 1 wt% of catalyst in the micro-batch closed system yielded 96% hydrocarbons with 93% *n*-(C_15_ + C_17_) selectivity within 60 min at 330 °C, 38.4 wt% FFA and 5% water content. An examination of the WCO under a series of FFA (0–20%) and water contents (0.5–20 wt%) indicated an enhanced yield of green diesel, and increased involvement of the deCOx mechanism. A high water content was found to increase the decomposition of triglycerides into FFAs and promote the DO reaction. The present work demonstrates that WCO with significant levels of water and FFAs generated by the food industry can provide an economical and naturally replenished raw material for the production of diesel.

## Abstract 2

Untreated waste cooking oil (WCO) with significant levels of water and fatty acids (FFAs) was deoxygenated over Co_3_O_4_–La_2_O_3_/AC_nano_ catalysts under an inert flow of N_2_ in a micro-batch closed system for the production of green diesel.

## Graphical abstract

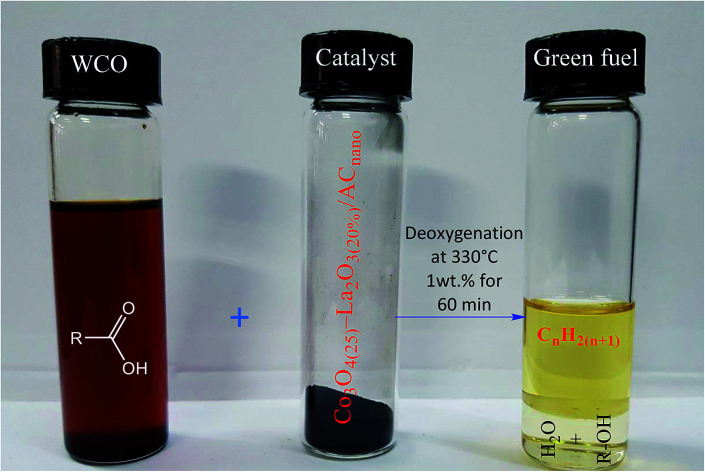

## 1. Introduction

A range of geopolitical, economic and environmental issues (*e.g.* global warming, diminishing petroleum deposits, increasing crude oil prices and demand for energy independence) have promoted recent interest in biofuels. Accordingly, fuels obtained from biomass (biofuels) are considered as a carbon-neutral and renewable substitute for fossil fuels. In particular, much attention has been paid to bio-oils, which can readily be obtained *via* the pyrolysis (thermal and catalytic cracking) of biomass. Nevertheless, the considerably low selectivity of this approach, which generates a range of unwanted products (primarily oxygenates), requires additional development. On the other hand, the well-established biofuel, which is known as biodiesel or fatty acid methyl esters (FAMEs), is composed of highly oxygenated compounds and suffer from several inevitable drawbacks such as poor storage stability and poor cold-flow behaviour.^1,2^ Consequently, elimination of oxygen-bonded compounds is necessary to achieve desirable diesel properties. Significantly, the high oxygen content of biodiesel and bio-oils gives rise to most of their disadvantages, including a lower heat content than that of conventional fossil fuels. Thus, the production of (oxygen-free) hydrocarbons from the catalytic deoxygenation (DO) of fatty acids and their derivatives has attracted interest. In principle, DO involves oxygen extraction from fatty acids and their derivatives *via* decarboxylation (–CO_2_) and decarbonylation (–CO, H_2_O) (deCOx) reactions under an H_2_-free atmosphere.

There are several solid acid catalysts containing metal sulfides, noble metals, metal phosphides, metal carbides, metals, and transition metal oxides with supporting materials reported for the DO reaction. Sulfonated acidic catalysts show high affinity toward the production of hydrocarbon fractions, but suffer from sulphur leaching and affect the quality of oil.^3,4^ Meanwhile, high acidity noble metal catalysts are costly,^5^ which make them unattractive. Medium acidic catalysts, such as metal phosphates and carbides, and mesoporous catalysts, such as SBA-15, MCM-41, and HMS catalysts, are generally selected for biofuel production since they exhibit low affinity toward the deactivation of the catalyst together with high DO activity.^6^ However, the complexity of their synthesis makes them unappealing.^7^ Thus, the drawbacks of the above mentioned catalysts encourage the exploration of non-sulphated, low-cost, and facile catalysts for the production of high-quality renewable fuel.

The use of metal oxides in the DO reaction has been continuously reported and proven to be selective towards the formation of hydrocarbon fractions.^8^ Common transition metal oxides (TMO) that are used in the DO process include Mn, Ni, Co, W, Mo, Cu, Fe and Zn. Among them, Co exhibits the highest rate of decarboxylation^9,10^ and formation of rich-paraffin species. The enhancement of decarboxylation by Co is realized through its excellent acidic–basic properties.^11^ Furthermore, Co also facilitates the formation of olefins (alkenes) *via* decarbonylation pathways, producing water as a by-product. However, water has a negative effect on Co, which is easily oxidized and deactivated. Recently, the incorporation of a Ca promoter in Co-supported SiO_2_–Al_2_O_3_ led to substantial catalyst stability and suggested the stability of Co relies on the incorporation of a basic metal, where the basic metal promoter leads to the inhibition of water production by lowering the affinity for the decarbonylation reaction.^12^ For instance, a report on the DO of waste cooking oil (WCO) *via* deCOx over acid-base catalysts (CaO–La_2_O_3_/AC_nano_ and Ag_2_O_3_–La_2_O_3_/AC_nano_) recently disclosed that the basic sites in La_2_O_3_ play key role in the removal of C–O-bonded species *via* decarboxylation, and thereby lead to greater enhancement of the DO activity and product selectively toward *n*-(C_15_ + C_17_) fractions.^3,12^ Furthermore, the basic sites of La_2_O_3_ suppress coke colonization. Recently, Benito and co-workers examined the effect of the addition of La_2_O_3_ to Ni/Al_2_O_3_, and observed that La_2_O_3_ promotion led to increased catalyst longevity, probably due to the oxygen donation from La_2_O_2_CO_3_ species to produce CO from carbon deposits.^13^ Even though a series of binary oxide CaO–La_2_O_3_ and Ag_2_O_3_–La_2_O_3_-supported AC_nano_ have been studied in details,^3,12^ their DO performance was unsatisfactory and rapidly deactivated by coke deposition. Based on previous findings, the incorporation of Co in La_2_O_3_/AC may be promising for the improvement in DO performance and the enhancement of anti-coking character. Furthermore, the use of Co_2_O_3_–La_3_O_4_ as a metal promoter on AC support has not been reported to date in the literature.

As is known, DO under an inert atmosphere is not beneficial for the formation of desired paraffins products. Green diesel consists of low mixtures of paraffin compounds, which are typically not beneficial for reducing NOx emission and possess a low cetane number. Furthermore, DO under an inert atmosphere prevents the production of a high mass of green diesel since the volatile liquids with non-condensable gases easily escape during the reaction. Thus, since the DO reaction atmosphere plays an important role in enhancing the formation of paraffins, in the present work, WCO was deoxygenized in a semi-batch inert flow reactor and micro-batch reactor over the binary oxide of Co_3_O_4_–La_2_O_3_ on AC_nano_ catalyst. The micro-batch reactor is a one-dimensional reactor (10 cm length × 0.8 cm diameter), providing rapid heat and high mass transfer rates.^14^ Although the micro-batch reactor has been successfully utilized for the continuous operation of fast and highly exothermic reactions and transformations involving toxic and explosive chemicals such as hydrogenation, alkylation, oxidation, and polymerization,^15–17^ this reactor system is rarely adopted in the DO reaction for the production of green diesel. Instead of varying the DO reactor set-up, the stoichiometric study of Co content (5–25 wt%), reaction time, catalyst loading, water and FFA content were explored. This study provides new insight into the interaction of Co_3_O_4_–La_2_O_3_ on AC_nano_ support and offer an alternative approach to curb coke deposition and improve the longevity of the catalyst during the DO reaction together with the formation of saturated hydrocarbon. The present work also provides new in-depth insight on the DO of WCO in inert and closed reaction systems.

## 2. Materials and methods

### 2.1 Materials

For the present study, the feedstock (palm oil-based WCO supplied by a restaurant at Serdang, Selangor, Malaysia) was used without purification. Table 1 summarises the characteristics of the crude WCO, including free fatty acid content and water content. The level of free fatty acids (18.4% by weight) is reflected by the total acid number (TAN) of 36.8 mg KOH g^−1^. The fatty acid content of the WCO included the saturated palmitic (45.68%), stearic (4.25%) and myristic acid (1.3%), together with the unsaturated oleic (40.19%) and linoleic acid (7.90%). Anhydrous silver nitrate (CoNO_3_, 99.99% pure) was supplied by Sigma Aldrich (UK), lanthanum nitrate hexahydrate (La (NO_3_)_3_·6H_2_O, 99.0% pure) was supplied Merck (Germany), phosphoric acid (H_3_PO_4_, 85.0–87.0% pure) was supplied by J. T. Baker (USA), and Walnut shells (*Juglans* sp.) were obtained from a market in Basra (Iraq). Liquid alkane and alkene standards (*n* = C_8_–C_20_) for gas chromatographic analysis were supplied by Sigma Aldrich and used without additional purification. The internal standard was 1-bromohexane. GC-grade *n*-hexane, purity >98%, supplied by Merck (Germany), was used for dilution. N_2_ gas with 99% purity was supplied by Linde Malaysia Sdn Bhd.

**Table 1:** Properties of the waste cooking oil (WCO) used as the reaction feedstock

Properties	Value
Density (g cm^−3^)	0.87
Viscosity (mm^2^ s^−1^)	4.85
Moisture content (% wt)	1–5
Acid value (mg KOH g^−1^)	36.81
FFA content (%)	18.40

**Fatty acid composition**	
Myristic acid, C_14_:0	1.93
Palmitic acid, C_16_:0	45.68
Stearic acid, C_18_:0	4.25
Oleic acid, C_18_:1	40.19
Linoleic acid, C_18_:2	7.95

### 2.2 Catalyst preparation

A two-step process and additional treatment with H_3_PO_4_ were chosen for the preparation of activated carbons with a prolate shape, surface functionality and high purity. Initially, 50 g of dried walnut shell powder with a particle size in the range of 1–200 mesh was subjected to pyrolysis in an N_2_ atmosphere at a heating rate of 5 °C min^−1^ up to 700 °C for 5 h. The sample was heated in a tube furnace using a ceramic vertical reactor. The carbonaceous product was activated by concentrated phosphoric acid at 158 °C for 12 h, then washed with hot water to pH = 7 and further dried in an oven overnight to form activated carbon (AC). The AC was then infused with 20 wt% La(NO_3_)·6H_2_O and 5–30 wt% Co(NO_3_) under continuous stirring for 6 h. The impregnation of the metal on the support was optimized *via* the vacuum impregnating procedure, and this was facilitated with a vacuum machine (Edwards RV12) at 6.3 × 10^−6^ mbar. Subsequently, a Stuart RE300DB rotary evaporator was employed for drying, under heating and reduced pressure for 3 h at 55 °C and −60 kPa, respectively. Subsequently, the AC-doped metals underwent calcination at 700 °C, which lasted for a 4 h period under an N_2_ flow. The metal-doped AC was denoted as Co_2_O_3(*x*)_–La_2_O_3(*y*)/_AC_nano_, where *x* = 5, 10, 15, 20, 25, and 30 wt%.

### 2.3 Material characterization

For the purpose of identifying the chemical composition and the state of dispersion of the metal-doped AC catalysts before and after the chemical reaction, X-ray diffraction (XRD) analysis was applied (Shimadzu, model XRD-6000). The Brunauer–Emmett–Teller (BET) technique was employed using an N_2_ adsorption/desorption analyser (Thermo-Finnigan Sorpmatic 1990 series) to obtain information on the surface area, and pore size and volume distribution of the catalysts. Typically, the catalyst sample was degassed at a temperature of 150 °C overnight to eliminate foreign gases and moisture from the surface of the catalysts. The analysis was performed at −196 °C, and the desorption and adsorption of nitrogen (N_2_) on the surface of the catalyst was performed in a vacuum chamber. Temperature-programmed desorption (TPD) was employed to examine the acidity and basicity of the catalysts. Here, two probe molecules, NH_3_ and CO_2_, were employed (TPD–CO_2_ and TPD–NH_3_, respectively). A Thermo Finnigan TPD/R/O 1100 instrument equipped with a thermal conductivity detector (TCD) was employed to conduct the examination. Typically, about 0.05 g of catalyst was pre-treated with an N_2_ gas flow, which lasted for 30 min at a temperature of 250 °C. This process was followed by exposing the catalyst to CO_2_ gas for adsorption within 1 h and flushing with N_2_ gas to remove excess CO_2_. Subsequently, CO_2_ desorption from the basic sites of the catalyst was identified by using the TCD under a flow of helium gas (30 mL min^−1^) in the temperature range of 50–900 °C. The temperature was kept constant for 30 min. NH_3_ adsorption/desorption was performed using similar steps as the TDP-CO_2_ method. An electron microscope (LEO 1455 VP) was employed to record field-emission scanning electron microscopy (FESEM) images, which was equipped with energy dispersive X-ray (EDX) (facilitated by a Rayny EDX-720 spectrometer) to determine the elemental composition of the catalyst samples (P, Co, La, O and C). X-ray photoelectron spectroscopy (XPS) was used to study the chemical states on the surface of the Co_2_O_3_–La_2_O_3_/AC_nano_ sample, and measurements were performed with a Microprobe PHI Quantera II under ultrahigh vacuum (UHV) conditions (base vacuum of ∼10^−8^ Pa) at room temperature. For the X-ray source, radiation of Mg Kα (*hν* = 1253.6 eV) was employed. Furthermore, thermal analysis using a thermogravimetry analyzer (TGA) (TGA 1000i, Instrument Specialists Inc, United States) was applied to identify the level of deposition of coke and carbon on the spent catalyst. Both the catalyst and spent catalyst were heated in the temperature range of 25–900 °C range (30 °C min^−1^) with an airflow of 40 mL min^−1^.

### 2.4 Catalytic deoxygenation of WCO

The DO of WCO was carried out in a 250 mL mechanically stirred semi-batch reactor. WCO (∼10 g) was placed in the reactor together with 0.5 wt% of catalyst, and prior to the initiation of the experiment, inert N_2_ gas was flushed into the reactor accompanied by continuous stirring of the mixture. The purpose of this process was to ensure that the O_2_ and moisture from the air was eliminated before heat was applied. Afterwards, the mixture was continuously treated with a constant flow of N_2_ (flow rate of 20 mL min^−1^) at atmospheric pressure to maintain the inert conditions for the DO reaction. The DO reaction was performed at 300 °C for 1 h. A cooler was used to facilitate the condensation of the deoxygenated products, where the condensates were collected in a vessel of the batch reactor. After completing each experiment, the mixture was cooled to room temperature. For comparison, a 10 cm length and 0.8 cm diameter stainless steel tube was used as a micro batch reactor closed system, where initially, the catalyst and WCO were mixed and placed in the micro-reactor and then the system was flushed using nitrogen gas for 10 min with a flow of 3 mL min^−1^ and then the valve was closed and the reactor heated at a heating rate of 50 °C min^−1^ to the desired temperature, and after the reaction was finished, the system was cooled using ice-cold water. The mixture of catalyst, char and product were separated using a centrifuge (3500 rpm for 10 min). The final deoxygenated liquid products were further analysed by using gas chromatography with a flame ionization detector (GC-FID), gas chromatography-mass spectrometry (GC-MS) and Fourier-transform infrared spectroscopy.

### 2.5 Water and FFA content effect

The water and FFA contents have a direct effect on the DO reaction. Fatty acids are known to be the primary composition during the DO reaction of triglycerides. Therefore, oleic acid was chosen as a model reactant. The water content in WCO will affect hydrolysis process of TG, where the TG molecules split into free fatty acids and propane. To vary the FFA content, the WCO used in this study consisted of average triglycerides of 92 wt%, free fatty acids of 6.6 wt%, and unknown impurities of 1.4 wt%. The feedstock was free of water. Additional water or free fatty acid (oleic acid) was mixed with the WCO to adjust the initial parameters for the reaction tests. The fatty acid composition of the WCO was evaluated according to the standard AOCS method (1997), F9a-44 (Table 1). The WCO consisted of C_14_–C_20_ fatty acids with the majority being unsaturated C_18_:1 (oleic acid) and saturated C_16_:0 (palmitic acid).

### 2.6 Product analysis

The reaction was analysed using an Agilent 7890A gas chromatograph (GC) fitted with a flame-ionization detector and an HP-5 capillary column (30 m × 0.25 mm × 0.33 mm). The sample (1 mL) was injected with a split ratio of 10 : 1. The carrier gas was nitrogen with a flow rate of 11 mL min^−1^. The injector temperature was 280 °C and the detector temperature was 300 °C. The oven was programmed to maintain a temperature of 40 °C for 4 min before ramping up to 280 °C at a rate of 10 °C min^−1^, maintaining this final temperature for 5 min. The products of the reaction were identified by comparing the retention times on the gas chromatograph with known standards and by examining the fragmentation patterns on an Agilent 5970 mass spectrometric (MS) detector. Calibration curves were used to provide quantitative analysis for each compound of interest. The determination of hydrocarbon yield (X) on the catalyst performance was evaluated by GC-FID using eqn (1) as follows:^18^1
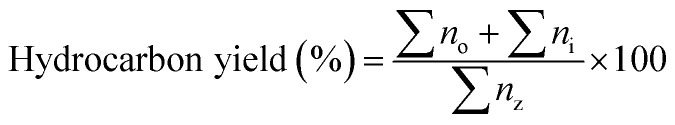
where *n*_o_ = peak area of alkenes (C_8_–C_20_), *n*_i_ = area of alkanes, and *n*_z_ = area of the product. The hydrocarbon selectivity (*S*) of the deoxygenated products was determined using eqn (2).2
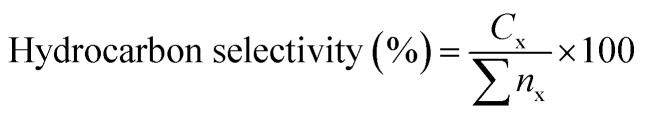
where *C*_x_ = peak area of the desired hydrocarbon fraction and *n*_x_ = area of hydrocarbons. The uncertainties were determined by repeated experiments and were reported as standard deviations, with each data point representing the mean average result obtained from a minimum of three separate experiments.

## 3. Results and discussion

### 3.1 Characterization of Co_3_O_4(*x*)_–La_2_O_3(*y*)_/AC_nano_ catalysts

**Fig. 1:**
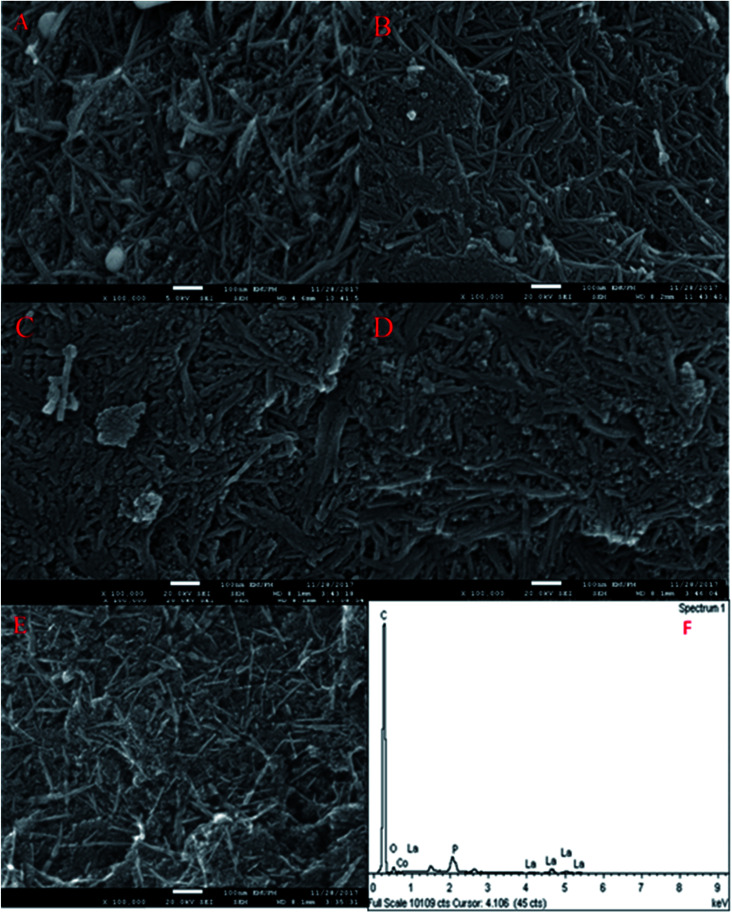
FESEM images of (A) Co_3_O_4(5%)_–La_2_O_3(20%)_/AC_nano_, (B) Co_3_O_4(10%)_–La_2_O_3(20%)_/AC_nano_, (C) Co_3_O_4(15%)_–La_2_O_3(20%)_/AC_nano_, (D) Co_3_O_4(20%)_–La_2_O_3(20%)_/AC_nano_, and (E) Co_3_O_4(25%)_–La_2_O_3(20%)_/AC_nano_ catalysts and (F) EDX spectra for Co_3_O_4(20%)_–La_2_O_3(20%)_/AC_nano_ catalyst.

The FESEM images of the nanosized walnut shell-derived AC catalyst were presented in our recent report.^3^ The result indicated the presence of nanocrystalline fibrous particles with a thickness in the range of 15–20 nm on the AC catalyst support. Interestingly, the FESEM images of the Co_3_O_4(*x*)_–La_2_O_3(*y*)_/AC_nano_ catalysts, where *x* = 5, 10, 15, 20, 25 wt% and *y* = 20 wt%, showed well-dispersed, needle-like particles with a long and thin slab of nano-structures (Fig. 1A–E). The particle length and diameter were within the range of 190–290 nm and 11–23 nm, respectively. A further increment in Co species led to the formation of compact micro-sized agglomerates. Based on the EDX analysis, the white spherical particles are attributed to Co species (identified by EDX analysis), which decreased in size and disappeared with an increase in the content of Co species contents. The change in morphology at different Co loading was due to the dopant effect, and thus resulted in aggregation.^19^ The needle-like structures were deformed as the Co aggregates grew excessively on the catalyst surface. Fig. 1F and Table 2 show the EDX analysis of the Co_3_O_4(*x*)_–La_2_O_3(*y*)_/AC_nano_ catalysts, which indicate the presence of C, O, P, La, and Co elements. Thus, the treatment of AC with H_3_PO_4_ resulted the presence of P,^20,21^ while the successful surface impregnation of La and Co was reflected by the presence of the corresponding elements in the EDX analysis. According to Table 2, the atomic ratio of Co : La in the catalysts was consistent with the expected (*x* : *y*) values of 5 : 20, 10 : 20, 15 : 20, 20 : 20 and 25 : 20. Overall, each of the Co_3_O_4(*x*)_–La_2_O_3(*y*)_/AC_nano_ catalysts had a maximum carbon content of >39%, which is expected to provide outstanding mechanical properties for both stability and thermal catalytic activity during DO reactions.

**Table 2:** Catalyst element composition determined by energy-dispersive X-ray spectroscopy (EDS)

Catalyst^a^	Element composition^b^ (%)				
	C	O	P	La	Co
Co_3_O_4(5)_–La_2_O_3(20%)_/AC_nano_	52.44	11.74	12.71	18.73	4.38
Co_3_O_4(10)_–La_2_O_3(20%)_/AC_nano_	49.37	10.46	9.27	19.60	11.30
Co_3_O_4(15)_–La_2_O_3(20%)_/AC_nano_	47.63	9.40	9.24	18.39	15.34
Co_3_O_4(20)_–La_2_O_3(20%)_/AC_nano_	43.16	8.43	10.21	19.23	18.97
Co_3_O_4(25)_–La_2_O_3(20%)_/AC_nano_	38.98	5.31	9.47	19.50	26.74

a Theoretical Co/La atomic ratio of catalyst.

b Experimental Co/La atomic ratio in the synthesized catalyst determined by EDX.

**Fig. 2:**
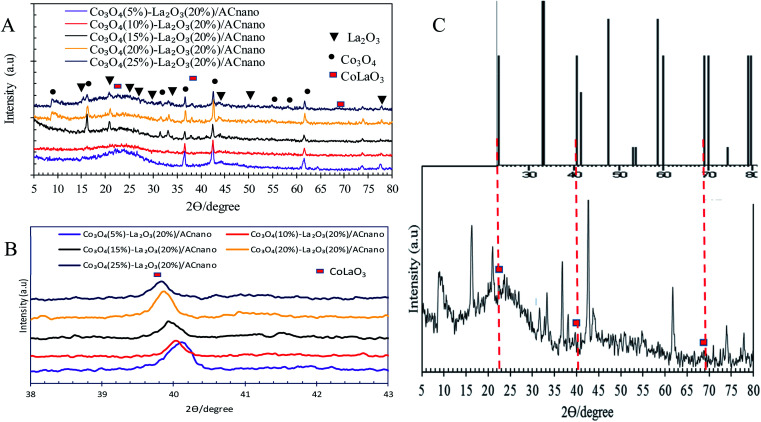
(A) XRD patterns of Co_3_O_4(*x*)_–La_2_O_3(20%)_/AC_nano_ catalysts, (B) bimetallic phase shifting and (C) bimetallic phase of CoLa_2_O_3_ of Co_3_O_4(25)_–La_2_O_3(20%)_/AC_nano_ catalyst.

Fig. 2A presents the XRD analysis of AC and Co_3_O_4(*x*)_–La_2_O_3(*y*)_/AC_nano_ catalysts. AC displayed a peak centred at 2*θ* = 25.0°, which indicates the presence of amorphous carbonaceous materials.^22^ In the case of the Co_3_O_4(*x*)_–La_2_O_3(*y*)_/AC_nano_ catalysts, the amorphous AC was altered to a highly crystalline state after the incorporation of the Co–La metals. Here, the XRD analysis revealed peaks at 2*θ* = 9.1°, 17.1°, 31.5°, 36.7°, 43.1°, 54.5°, 59.3° and 62.7°, which correspond to the Co_3_O_4_ phase (JCPDS File no. 00-001-1152).^23^ The XRD peaks at 2*θ* = 14.1°, 18.1°, 25.3°, 26.6°, 29.6°, 39.2°, 46.6°, 54.6°, and 77.6° are attributed to the La_2_O_3_ phase (JCPDS File no. 00-037-1497).^24,25^ The highly crystalline nature of the Co_3_O_4(*x*)_–La_2_O_3(*y*)_/AC_nano_ catalyst is due to the intercalation of La and Co ions in the AC matrix.^26,27^ Upon increasing the Co dosage (from 5 to 25 wt%), the occurrence of bimetallic lanthanum cobalt oxide phases (CoLaO_3_) at 2*θ* = 23.3°, 40.2° and 69.9° (JCPDS File no. 00-006-0491)^28,29^ (Fig. 2B and C) evidenced the effective integration of the bimetallic (Co–La) structure into the AC_nano_ catalyst. Moreover, the bimetallic CoLaO_3_ peaks corresponding to the (110)^1^ plane gradually shifted to a lower angle with an increase in the Co content. This phenomenon indicates that more Co atoms were located deep in the La_2_O_3_ lattice with an increase in the Co content, indicating that the higher the Co content, the higher the amount of Co–LaO_3_ solid solution is formed.^2^ The homogeneous substitution of Co with La easily occurred because the radius of La^3+^ (1.16 Å) is larger than that of Co^2+^ (0.65 Å). Also the incorporation of Co-rich species in the Co_3_O_4(*x*)_–La_2_O_3(*y*)_/AC_nano_ catalyst promoted the dispersion of CoLaO_3_ and reduced the crystallinity of CoLaO_3_.^3,4^ The present finding is consistent with the EDX results, where the oxygen content was reduced by 54% after the incorporation of 25 wt% of Co species (Table 2). Although the atomic radius of oxygen (1.67 Å) is larger than that of Co (0.65 Å), excess replacement by Co increased the size of the unit cell for CoLaO_3_.^11^ The increment in the average crystallite size for the Co_3_O_4(*x*)_–La_2_O_3(*y*)_/AC_nano_ catalyst with an increase in Co dosage is depicted in Table 3.

**Table 3:** Physicochemical properties of the Co_3_O_4(*x*)_-La_2_O_3(*y*)_/AC_nano_ catalysts

Catalyst	XRD^a^	BET^b^		TPD-NH_3_^c^		TPD-CO_2_^c^	
	Crystallite size^a^ (nm)	Surface area^b^ (m^2^ g^−1^)	Pore volume^b^ (cm^3^ g^−1^)	NH_3_ desorption temperature^c^ (°C)	Amount of NH_3_ adsorbed^c^ (μmol g^−1^)	CO_2_ desorption temperature^c^ (°C)	Amount of CO_2_ adsorbed^c^ (μmol g^−1^)
Co_3_O_4(5%)_–La_2_O_3(20%)_/AC_nano_	38.40	668.53	0.64	127/730/806	148.74/2584.76/19 266.00	635	3289.72
Co_3_O_4(10%)_–La_2_O_3(20%)_/AC_nano_	44.49	634.76	0.61	130/634/800	214.39/1019.17/16 247.95	546	3510.07
Co_3_O_4(15%)_–La_2_O_3(20%)_/AC_nano_	46.23	514.36	0.57	138/819/842	249.84/1191.16/18 843.94	906	3865.96
Co_3_O_4(20%)_–La_2_O_3(20%)_/AC_nano_	48.41	501.79	0.52	135/609/831	254.21/4042.96/26 165.70	939	5292.06
Co_3_O_4(25%)_–La_2_O_3(20%)_/AC_nano_	53.18	486.13	0.41	140/713/832	251.25/713.16/4400.43	901	3272.65

a The crystallite size was determined from the highest intensity line broadening of the XRD peak at 2*θ* = 43.1°.

b BET surface area.

c NH_3_/CO_2_ desorption peak for all the catalysts.

Table 3 lists the BET surface area for the Co_3_O_4(*x*)_–La_2_O_3(20)_/AC_nano_ catalysts with different Co : La ratios. The results indicate a decrease in the BET surface area from 668 to 486 m^2^ g^−1^ with an increase in Co dosage from 5 to 25 wt%. The reduction in surface area with an increment in the Co content is due to the destruction of the needle-like structure together with the formation of excessive small aggregates (see FESEM image), which covered the active sites of the catalyst surface.^30–32^ Moreover, the pore volume of the Co_3_O_4(*x*)_–La_2_O_3(20)_/AC_nano_ catalyst decreased from 0.64 to 0.41 nm with an increase in Co content. These observations are attributed to the incorporation of excess active metals (Co and La) in the pores of the AC support, which slightly blocked the porous structure.^24^

**Fig. 3:**
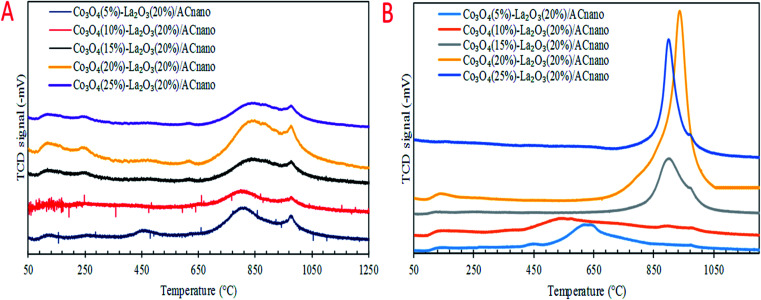
(A) TPD-NH_3_ and (B) TPD-CO_2_ profiles for the Co_3_O_4(*x*)_–La_2_O_3(20)_/AC_nano_ catalysts.

Previous work reported that acidic sites are necessary for enhancing the C–O and C–C bond cleavage *via* decarboxylation/decarbonylation and cracking pathways.^33^ Besides acidic sites, basic sites play an important role in promoting decarboxylation and suppressing the formation of coke by reducing the deactivation rate of acidic catalysts.^34,35^ Hence, the perfect and ideal DO catalyst can be achieved *via* the co-existence of basic and acidic sites. Thus, the acidity and basicity profiles for the Co_3_O_4(*x*)_–La_2_O_3(*y*)_/AC_nano_ catalyst were quantified by temperature-programmed desorption NH_3_ (TPD-NH_3_) and CO_2_ desorption (TPD-CO_2_) analyses, and the results are displayed in Fig. 3A and B and Table 3. The TPD–NH_3_ profiles in Fig. 3A display two distinct NH_3_ desorption peaks at temperatures of <150 °C and >500 °C, which are associated with the weak and strong acidic sites, respectively.^36^ An examination of Table 3 reveals that the increment of Co doping from 5 to 20 wt% induced the generation of strong acidic sites, which suggests that the presence of Co-rich species enhances the strength of the acidic sites. However, a dramatic decrease in the concentration of strong acid sites was observed with a further increment in Co content to 25 wt%. This can be attributed to the structural changes caused by the high Co content, which led to damage of the mixed oxide structure (Fig. 1E). The highest acidity distribution (30 208.66 μmol g^−1^) was observed at a Co content of 20 wt% with the maximum increase of approximately 28% over the Co_3_O_4(5%)_–La_2_O_3(20%)_/AC_nano_ catalyst. The weak acidity indeed showed negligible changes (148.74–254.21 μmol g^−1^) for all the Co_3_O_4(*x*)_–La_2_O_3(*y*)_/AC_nano_ catalysts. The basicity (TPD–CO_2_) profiles of the various Co_3_O_4(*x*)_–La_2_O_3(*y*)_/AC_nano_ catalysts are presented in Fig. 3B and Table 3. Each catalyst displayed the main desorption peak at a temperature of >500 °C, which is attributed to the interaction of CO_2_ with the strong basic sites of the catalyst. Similar to the trend on the acidity profile, the strong basic site distribution was found to increase with an increase in Co dopant up to 20 wt%. It is generally acknowledged that the basicity content will improve with an increase in Co loading.^37–39^ This is due to the synergistic effect between Co and La on the activated carbon surface.^40–43^ However, excess Co dopant covers the active sites of La, and thus provides lower basicity. Consistent with this, a reduction in the distribution of basic sites (5292.06 μmol g^−1^) was found on the Co_3_O_4(25)_–La_2_O_3(20)_/AC_nano_ catalyst. In summary, the Co_3_O_4(20%)_–La_2_O_3(20%)_/AC_nano_ catalyst exhibited the highest CO_2_ desorption peaks at *T*_max_ = 939 °C with the maximum basic site distribution (5292.06 μmol g^−1^). Considering the acidity and basicity, an excess of Co dopant (25 wt%) results in high saturation of the Co-active sites, consequently hindering the active sites of La. Since, the capacity to instigate C–O cleavage is directly related to a high distribution of acid and basic active sites, it is speculated that elevated Co dosages of >20% will result in lower DO activity. Based on the result obtained, it is expected that the most effective ratio for converting WCO to hydrocarbon-like structures *via* DO is the CoO_(20%)_–La_2_O_3(20%)_/AC_nano_ catalyst.

Since the Co_3_O_4(20%)_–La_2_O_3(20%)_/AC_nano_ catalyst showed superior acid-base active sites, a detailed study on its surface was performed *via* XPS analysis. Fig. S1A–E† reveal the deconvolution peaks at a binding energy (BE) of 284.7–289.8 eV (C), 531.8–533.1 eV (O) and ∼133.7–135.7 eV (P) together with the characteristic peaks for La 3d_5_ and Co 2p_3_. The O 1s spectra for the Co_3_O_4(20%)_–La_2_O_3(20%)_/AC_nano_ catalyst present two peaks with the BE values of 529.8 eV, 531.8 eV and 533.1 eV (Fig. S1B†). The XPS peak at 529.8 eV is attributed to the lattice O^2−^ species, O_2_^2^/O, whereas the peak at 531.6 eV originated from the hydroxyl species, OH^−^. The highest BE level peak at 533.10 eV is assigned to molecular water adsorbed on the surface of the catalyst.^44,45^ This implies that the lanthanum and cobalt are in the form of oxides on the catalyst surface. It noteworthy to mention that the O 1s region showed the lowest BE peak compared to other elemental species, which are known to be less electron-rich oxygen species.^44^ This is in agreement with surface atomic ratio data shown in Table 4, where only 6% O was detected on the Co_3_O_4(20%)_–La_2_O_3(20%)_/AC_nano_ catalyst. This result is also in accordance with the FESEM-EDX result, where only ∼5% O was detected (Table 2). Thus, this finding confirms that the Co_3_O_4(20)_–La_2_O_3(20)_/AC_nano_ catalyst is comprised mainly of bimetallic Co–La alloy supported on AC_nano_. The carbon in the Co_3_O_4(20)_–La_2_O_3(20)_/AC_nano_ catalyst was detected in the C 1s spectra (Fig. S1C†). Four distinct peaks were obtained at BE of 284.7, 285.8, 286.9 and 289.8 eV. These peaks are attributed to C–C, C–O, C=O and C(O)O, respectively, demonstrating that three types of functional groups were incorporated during the synthesis of the catalyst. The value for C 1s belonging C–C was the highest (56%), implying that the AC_nano_ support is mainly composed of carbon-based material, which was connected by C–C bonds. The total C content was relatively high (45%), and thus enhanced the thermally stability of the catalyst at high reaction temperatures.^46^ Referring to Fig. S1D,† the BE for the deconvolution curves of Co 2p were observed in the range of 781.9 eV (Co 2p_3/2_) to 797.8 eV (Co 2p_l/2_). This is evidence of the existence of cobalt metal on the catalyst surface. The significant deconvolution curves of Co 2p suggest that the cobalt metal was well distributed on the external AC surface, and it is speculated that the improvement in DO catalytic activity is due to the greater cobalt dispersion on the AC.^47^ Fig. S1E† indicates deconvolution of the high-resolution P 2p spectra into a single peak, with binding energies of 133.7 eV for P 2p_3/3_ and 135.3 eV for P 2p_1/2_. These observations are linked to the presence of PO_4_^3−^, which was derived from the phosphate precursor of H_3_PO_4_ used in the chemical activation of the walnut shell.^25^ Fig. S1F† indicates the presence of two regions displayed by La 3d_5_ in lanthanum oxide (838.3 eV and 835.2 eV), which are associated with La^3+^ species on the catalyst surface.^48^ Notably, the XPS results revealed a significant bonding interaction between Co^2+^ and La^3+^ in terms of a shift in the binding energies of Co 2p_3/2_ (from 779.2 (Co_3_O_4_, standard) to 781.7), and a shift in the binding energy of La 3d_5_ (from 834.5 eV (La_2_O_3_, standard) to 835.6 + 0.1 eV).^44,49^ The shift in bond energy is also due to the formation of the bimetallic (La–Co) phase. The metallic bonding of La and Co was substantiated by the XRD results (refer to XRD).

**Table 4:** XPS peak deconvolution result in %, peak position, FWHM and surface concentration for the Co_3_O_4(20%)_–La_2_O_3(20%)_AC_nano_ catalyst

State of element	O 1s			C 1s				Co 2p	La 3d_5_	P 2p
Atomic percent (at%)	6.1			45.2				18.8	19.9	9.9
	O 1s. cf1	O 1s. cf2	O 1s. cf3	C–O	C–C	C=O	C(O)O	Co 2p_1/2_		P 2p_3/2_
Peak position (eV)	529.81	531.83	533.10	285.81	284.70	286.90	289.81	797.80	835.60	133.72
FWHM	1.42	1.80	196	1.42	1.26	1.57	1.55	2.96	—	2.12
Concentration (%)	16.35	56.55	27.09	27.98	55.69	10.87	5.47	—	—	—

### 3.2 DO of WCO over Co_3_O_4(*x*)_–La_2_O_3(*y*)_/AC_nano_ catalysts and effect of reaction atmosphere

**Fig. 4:**
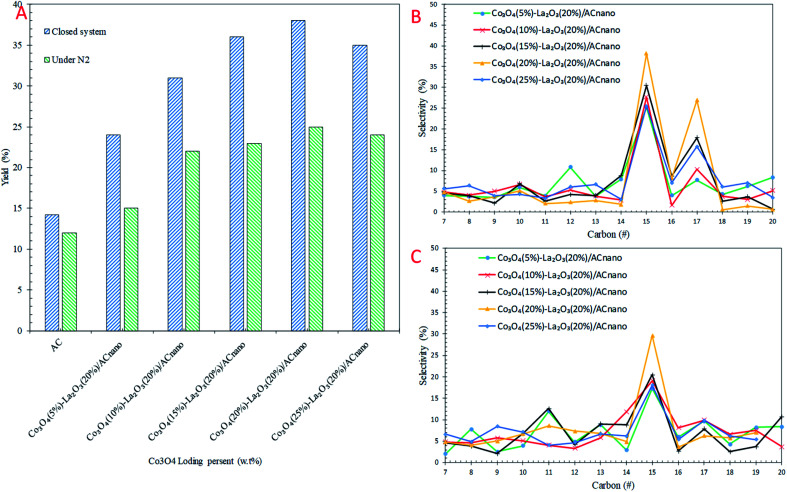
(A) Effect of Co concentration from 5–25 wt% on (A) hydrocarbon yield, (B) selectivity toward C_8_–C_20_ of the micro-batch closed system and (C) selectivity toward C_8_–C_20_ under inert conditions with the reaction conditions of 0.5 wt% of catalyst loading, 1 h at 300 °C.

The catalytic DO of WCO over the Co_3_O_4(*x*)_–La_2_O_3(*y*)_/AC_nano_ catalysts was conducted at a constant temperature (300 °C), for 1 h, with 0.5 wt% catalyst loading under two different conditions: (i) N_2_ flow and (ii) closed system. The detailed GC-FID results are shown in Fig. 4. For comparison, a blank experiment was carried out over the AC_nano_ support. The results showed that the deoxygenized liquid products produced from both reaction systems were composed of saturated and unsaturated hydrocarbon fractions in the range of C_8_–C_20_ (Fig. 4A). Notably, AC_nano_ had lower activity for the DO of WCO than the Co_3_O_4(*x*)_–La_2_O_3(20%)_/AC_nano_ catalysts. The DO profile showed that a low hydrocarbon yield (12–14%) was achieved from AC_nano_ catalysing DO in both reaction systems. However, when the Co_3_O_4(20%)_–La_2_O_3(20%)_/AC_nano_ catalyst was applied, high activity was observed with a hydrocarbon yield in range of 25–38%. The yields of liquid hydrocarbon were significantly increased in the order of Co_3_O_4(20%)_–La_2_O_3(20%)_/AC_nano_ > Co_3_O_4(15%)_–La_2_O_3(20%)_/AC_nano_ > Co_3_O_4(25%)_–La_2_O_3(20%)_/AC_nano_ > Co_3_O_4(10%)_–La_2_O_3(20%)_/AC_nano_ > Co_3_O_4(5%)_–La_2_O_3(20%)_/AC_nano_ > AC_nano_. The hydrocarbons yield increased from 33–65% to 27–35% as the Co dopant increased from 5 to 20 wt% and reduced for 25 wt% Co concentration.

Theoretically, the WCO was composed majority of ∼46% C_16_ and ∼52% C_18_ fatty acids, which were further deoxygenized *via* decarboxylation/decarbonylation (deCOx) pathways to produce hydrocarbon fractions mainly of *n*-C_15_ and *n*-C_17_. Fig. 4B and C shows the carbon distribution of the deoxygenated liquid product obtained from both DO systems. Considering that *n*-(C_15_ + C_17_) was significantly found among the hydrocarbon products in all cases, it is conceivable that the *n*-(C_15_ + C_17_) obtained resulted from the decarbonylation (deCOx) synthesis pathway. As the Co content increased, the deCOx rate increased and resulted in formation of *n*-(C_15_ + C_17_) fractions.^50^ In addition, the total selectivity of *n*-(C_15_ + C_17_) was reduced (∼29–41%) when 25 wt% of Co catalysed the reaction. Although some studies acknowledged that high deCOx activity is dominated by catalysts with rich weak + medium acidic sites (TPD-NH_3_ desorption temperature <500 °C),^25,51,52^ it was difficult to correlate this finding since the weak acid obtained from all the catalysts was close in value (Table 3). The high hydrocarbon yield (25–38%) and *n*-(C_15_ + C_17_) fractions (36–65%) over the Co_3_O_4(20%)_–La_2_O_3(20%)_/AC_nano_ catalyst suggest that the DO activity *via* deCOx pathways was strongly affected by the surface density of strong acid and basic sites. This indicates that a higher degree of DO occurs over highly acidic and basic catalysts. The pronounced effect of acid-base characteristics in promoting the DO reaction is consistent with our previous investigations. The DO of WCO on Ag_2_O–La_2_O_3_/AC_nano_ and CaO–La_2_O_3_/AC catalysts suggested that deCOx is favoured by the existence of a large amount of acid-based sites.^3,12^

As expected there was a great difference in the hydrocarbon fraction distribution between the DO reaction using the micro-batch closed system and reaction under N_2_ flow. The DO of WCO under an N_2_ flow gave a low hydrocarbon yield (25%) and also poor deCOx activity with only 36% *n*-(C_15_ + C_17_) selectivity. Meanwhile, the *n*-(C_15_ + C_17_) selectivity was found to increase significantly in the micro-batch closed system, with 38% hydrocarbon yield and 68% *n*-(C_15_ + C_17_) selectivity. This indicates that the deCOx reaction was promoted by the catalytic reaction under the high pressure built-up in the micro-batch closed system. In addition, this suggests competitive activation of the active sites by the high pressure DO system, which led to an enhancement in the deCOx reaction. The low selectivity toward *n*-(C_15_ + C_17_) was prominent for DO catalysed by Co_3_O_4(*x*)_–La_2_O_3(*y*)_/AC_nano_ under an N_2_ flow, which is due to the formation of rich oxygen-containing compounds (oxygenates and CO_2_/CO gases) during the ineffective DO reaction. These oxygenates can act as poisons by strongly adsorbing on the catalyst surface and deactivate the active sites of the catalyst.

**Fig. 5:**
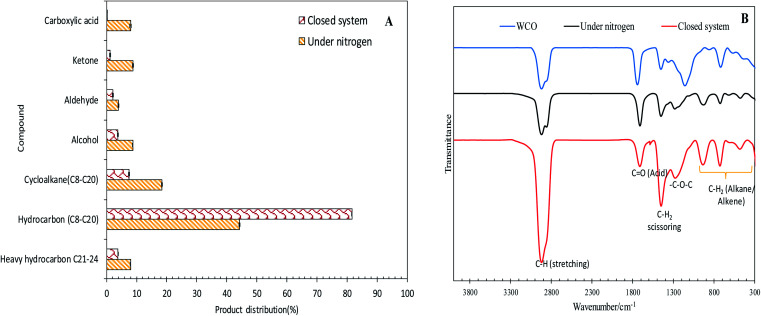
(A) Product distribution of deoxygenated liquid product analysed by GC-MS and (B) FTIR spectra of the deoxygenized liquid product under the reaction conditions of Co_3_O_4(20%)_–La_2_O_3(20%)_/AC_nano_, 0.5 wt% catalyst loading, 1 h reaction time, and 300 °C reaction temperature.

The efficacy of WCO DO under the closed and N_2_ flow systems over the Co_3_O_4(20%)_–La_2_O_3(20%)_/AC_nano_ catalysts was further confirmed by GCMS, as shown in Fig. 5A. It was observed that the DO of WCO in the micro-batch closed system produced a hydrocarbon fraction two times larger than that in the N_2_ flow system. Interestingly, the peaks for carboxylic acids could not be observed in the deoxygenated liquid product from the micro-batch closed system, where in contrast the N_2_ flow system produced >8% carboxylic acid. Moreover, the amount of other oxygenate species such as ketones, aldehydes and alcohols was remarkably reduced (7.13%) in the micro-batch closed system. Thus, it can be concluded that the Co_3_O_4(20%)_–La_2_O_3(20%)_/AC_nano_ catalyzed DO in the micro-batch closed system offers successive cracking reactions *via* C–O cleavage, producing rich hydrocarbon fuel mixtures. The FTIR analysis was performed to study the chemical functional groups of the WCO (feedstock) and deoxygenized liquid products (Fig. 5B). The FTIR spectrum of WCO showed absorption bands at 2915 cm^−1^ (–CH), 1692 cm^−1^ (–C=O) stretching, 1447 cm^−1^ (–CH_2_) scissoring, 1285 cm^1^ (–C–O–C) and 726 cm^−1^ (–(CH)_*n*_– bending for alkane).^3^ The FTIR results for WCO and the liquid deoxygenated product show that all the spectra were normalized by the intensity of the absorption band centered at 2753–3000 cm^−1^ (CH stretching, aliphatic). It is noteworthy that the liquid deoxygenated products showed a significant intensity reduction for the absorption band at 1692 cm^−1^, which belongs to C=O (fatty acid), and the absence of C–O–C (from carbonyl group in WCO) absorption features at 1285 cm^−1^. This result indicates the removal of oxygen species *via* deCOx pathways.^53,54^ The DO close system exhibited a significantly lower intensity for the C=O and C–O–C peaks compared to DO in the N_2_ flow system under the same experimental conditions.

### 3.3 Optimization studies

**Fig. 6:**
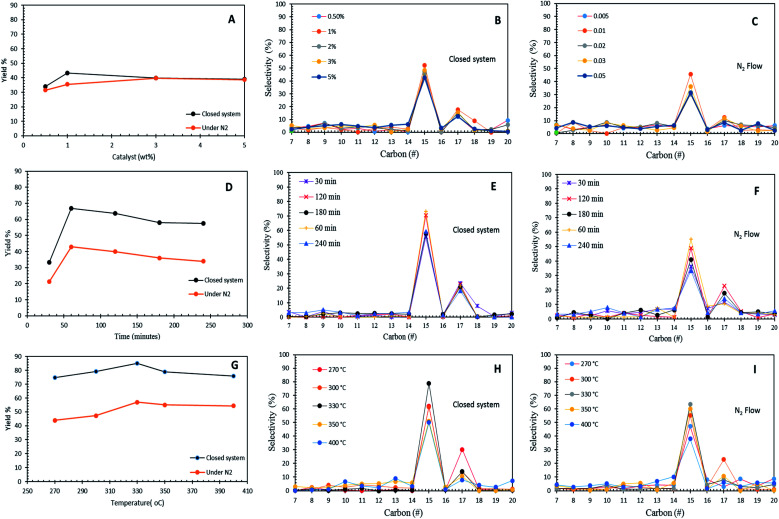
Optimization studies of WCO, (A–C) DO reactions of Co_3_O_4(20%)_–La_2_O_3(20%)_/AC_nano_ catalyst affected by catalyst amount; reaction conditions: 300 °C and 1 h. (D–F) DO reactions of Co_3_O_4(20%)_-La_2_O_3(20%)_/AC_nano_ catalyst affected by reaction time; reaction conditions: 1 wt% catalyst loading and 330 °C. (G–I) DO reactions of Co_3_O_4(20%)_–La_2_O_3(20%)_/AC_nano_ catalyst affected by reaction temperature; reaction conditions: 1 wt% catalyst loading, 1 h for 400 rpm under an N_2_ flow and micro-batch closed system.

The effects of the Co_3_O_4(20%)_–La_2_O_3(20%)_/AC_nano_ catalyst loading (0.5 wt% to 5 wt%) towards hydrocarbon yield and product selectivity at 300 °C temperature and 60 min reaction time under inert conditions and in the micro-batch closed system with a stirring speed of 400 rpm are shown in Fig. 6A–C. The results show that the DO activity increased with an increment in catalyst loading within the range of 0.5 wt% to 1 wt%, which yielded 43% and 35% of hydrocarbons with 69% and 47% *n*-C_15_ + *n*-C_17_ selectivity for the reaction using the micro-batch closed system and under N_2_ flow, respectively. This finding is possibly due to the fact that the increase in the catalyst loading enhanced the exposure of active sites for the accessibility of the feedstock in the DO reaction.^55^ However, a further increase in the catalyst loading to >1 wt% resulted a reduction in hydrocarbon yield to <40% and *n*-C_15_ + *n*-C_17_ selectivity to <43% in both DO systems. This is due to the occurrence of parallel or secondary reactions, such as polymerization pathways. The excess active sites due to a high catalyst loading increased the rapid-rate reaction on the catalyst surface, which simultaneously resulted in shortening of the catalyst life span by coke formation caused by polymerization reaction.^56^ In the present study, 1 wt% catalyst loading was observed to be optimal for the catalytic DO reaction.

Fig. 6D–F show the effect of reaction time in the range of 30 min to 240 min with 1 wt% Co_3_O_4(20%)_–La_2_O_3(20%)_/AC_nano_ catalyst loading at a temperature of 300 °C in the micro-batch closed system and inert N_2_ flow condition. The results showed that the DO activity depends on the reaction time. As the reaction time increased from 30 min to 60 min, the hydrocarbon yield increased from 33% to 66% and 21% to 39% for DO in the micro-batch closed system and inert condition, respectively. Meanwhile, the closed and inert N_2_ flow system also showed an increment of *n*-C_15_ + *n*-C_17_ selectivity from 79% to 94%, and 47% to 58%, respectively. Further prolonging the time to 240 min led to a slight reduction in the hydrocarbon yield from 66% to 57% and 39% to 34%, and the *n*-C_15_ + *n*-C_17_ selectivity was reduced to >77% and 47%, respectively. The insignificant changes in the hydrocarbon yield and *n*-C_15_ + *n*-C_17_ selectivity indicate that DO *via* deCOx reaction was highly unaffected in this time range. The lowest catalytic activity was observed at the longest reaction time (240 min), which is because unfavourable side reactions (*i.e.*, polymerization) occur during a longer reaction time. These side reactions accelerated the deactivation of the catalyst since the polymerization process generates coke on the catalyst. Coke formation reduces the effectiveness of the catalyst and leads to the accumulation of different compounds on the catalyst surface, covering the active sites, and thus suppressing the reaction at the catalytic centres of the catalyst.^57^

The effect of reaction temperature in the DO of WCO was investigated in the temperature range of 270 °C to 400 °C, with a Co_3_O_4(20%)_–La_2_O_3(20%)_/AC_nano_ catalyst loading of 1 wt%, and reaction time of 60 min in the micro-batch closed and inert N_2_ flow system with a stirring speed of 400 rpm (Fig. 6G–I). The DO activity increased with an increase in temperature from 270 °C to 330 °C. Both DO systems showed a similar trend of activity, whereby the highest catalytic activity was found at a temperature of 330 °C. This proves that Co_3_O_4(20%)_–La_2_O_3(20%)_/AC_nano_ is thermally stable since it is capable to withstand at a high temperature. A further increase in temperature to >330 °C led to the retardation of catalytic activity with a lower hydrocarbon yield and *n*-C_15_ + *n*-C_17_ selectivity. The high reaction temperature enhanced the occurrence of cracking *via* C–C scission in the triglycerides and *n*-C_15_ + *n*-C_17_ fractions into lighter fractions.^58^ Therefore, the formation of lighter fractions (*i.e.*, gaseous products) and gasoline fraction (*i.e.*, C_8_–C_12_) simultaneously reduced the hydrocarbon yield and *n*-C_15_ + *n*-C_17_ selectivity. In this study, the optimum reaction temperature in the N_2_ flow system was achieved at 330 °C, 1 wt% catalyst within 60 min, with 52% hydrocarbon yield and 71% *n*-C_15_ + *n*-C_17_ selectivity. On the other hand, the optimum catalytic DO (hydrocarbon yield = 85%, *n*-C_15_ + *n*-C_17_ = 93%) was achieved within 60 min and 1 wt% catalyst loading at 330 °C in the micro-batch closed system.

### 3.4 Effect of water and FFA content in DO of WCO

Other important factors affecting the DO activity are water and FFA content in the feedstock. It was reported in a previous study that the addition of water to the DO feedstock produced three beneficial effects: (1) low acidic and other low-value oxygenated components, (2) prevent the beneficial components that are partially miscible in water to not easily be excluded from DO; and (3) in particularly strong catalysts, water regulates the catalyst site interactions with the feedstock components.^59^ The investigation of the content of water and FFA on the DO of WCO over the Co_3_O_4(20%)_–La_2_O_3(20%)_/AC_nano_ catalyst was performed using the optimum parameters of 1 wt%, 60 min at 330 °C in the micro-batch closed system. All the obtained results are displayed in Fig. S2A–D.† Some publications suggest the negative impact of water towards DO activity, where water results in the modification of the external catalyst surface, simultaneously leading to the loss of some active metals species, and reduces the catalytic DO activity. Interestingly, the impact of water on the DO of WCO over the Co_3_O_4(20%)_–La_2_O_3(20%)_/AC_nano_ catalyst showed a positive effect (Fig. S2A and B†). In the present study, increasing the water content from 1 wt% to 5 wt% resulted in an increase in the hydrocarbon yield from 82% to 88%, while the *n*-C_15_ + *n*-C_17_ selectivity increased from 90% to 93%. This indicates that the addition of water to WCO facilitated the C–O cleavage of fatty acids *via* the deCOx reaction. The hydrocarbon yield and selectivity reached ∼88% and 93% *n*-C_15_ + *n*-C_17_ when the water content was >5 wt%, respectively. This indicates that the cobalt and lanthanum supported on AC_nano_ were chemically and thermally stable at high temperature even with a large amount of water in the feedstock.

Further study on the effect of FFAs towards the DO reaction was performed. Since the WCO consisted mainly of C_18_ of fatty acids, oleic acid (C_18_) was chosen as a model reactant. The FFA% of the WCO was controlled by further adding commercial oleic acid as a representative natural FFA. The FFA% for the feedstock mixture (WCO + oleic acid) used was in the range of 18% to 38% (Fig. S2C and D†). It was found that all the feedstocks were effectively deoxygenized *via* the selective deCOx reaction with a higher percentage of hydrocarbon fraction of >93%, and the product was predominantly selective toward *n*-C_15_ + *n*-C_17_ fractions. The total hydrocarbons was found to increase from 88% to 96% with an increase in FFA content from 18% to 38%. In contrast, the value of *n*-C_15_ + *n*-C_17_ selectivity was reduced to 84% from 94%. The results suggest that an FFA-rich feed encourages C–C cleavage rather than C–O cleavage. Evidently, *n*-C_9_, *n*-C_12_ and *n*-C_13_ were found to be prominent in the DO of the FFA-rich feedstocks (FFA > 23%). It has been reported that a higher content of FFA in the feedstock will lead to a decrease in DO activity and favour the cracking reaction. Overall, the DO of the FFAs–WCO mixtures was still favorable toward C–O cleavage and produced rich diesel-like fuels. This suggests the Co_3_O_4(20%)_–La_2_O_3(20%)_/AC_nano_ catalyst is highly adaptable to water–FFA conditions in the DO process, and thus it can be applied for other realistic feedstocks.

### 3.5 Reusability of Co_3_O_4(20%)_–La_2_O_3(20%)_/AC_nano_ catalyst

The reusability profile of the Co_3_O_4(20%)_–La_2_O_3(20%)_/AC_nano_ catalyst was investigated *via* DO reaction in the micro-batch closed system condition under the conditions of 1 wt% catalyst at 330 °C within 60 min (Fig. S3A†). Upon the completion of each cycle, the catalyst was reactivated by simple washing with hexane and reused for the next cycle. The results showed that the Co_3_O_4(20%)_–La_2_O_3(20%)_/AC_nano_ catalyst performed steadily for eight consecutive runs, maintaining the hydrocarbon yield at >72% with >80% selectivity of C_15_ and C_17_ products (Fig. S3A and S4†). This indicates that the Co_3_O_4(20%)_–La_2_O_3(20%)_/AC_nano_ catalyst possesses mechanical properties and chemical stability. Furthermore, the elemental study indicated a negligible amount of La^3+^ and Co^4+^ species leached from the Co_3_O_4(20%)_–La_2_O_3(20%)_/AC_nano_ catalyst during each reaction. The comparison study show that the leached La^3+^ and Co^4+^ in the liquid product of the fresh and 8^th^-run catalyst gradually increased from 1 ppm to 3 ppm and 1 ppm to 4 ppm, respectively. Hence, the loss in DO activity is due to the dissolution of the active metals during continuous runs. Furthermore, the leaching level is within the maximum range of the EN 12662 Standard Specification for Diesel Fuel Oils contamination content (24 mg L^−1^),^60^ which confirms that the Co_3_O_4(20%)_–La_2_O_3(20%)_/AC_nano_ catalyst shows potential leaching resistance and exhibits good stability.

The metal dispersion states and chemical composition study for the Co_3_O_4(20%)_–La_2_O_3(20%)_/AC_nano_ catalyst (fresh and spent catalysts) were determined by XRD and TGA analysis (Fig. S3B and C†), respectively. The XRD analysis showed that the spent catalysts exhibited similar bimetallic active CoLaO_3_ phases with higher crystallinity at 2*θ* = 23.3°, 40.2° and 69.9° (JCPDS File no. 00-006-0491). The crystallite size of the Co_3_O_4(20%)_–La_2_O_3(20%)_/AC_nano_ particles evaluated at 2*θ*: 43.1 showed a minor change in the crystallite size of the spent catalyst (Fig. S3B†). The TGA analysis was performed to examine the extent of coke formation during the DO of WCO using the Co_3_O_4(20%)_–La_2_O_3(20%)_/AC_nano_ catalyst. The results showed a decomposition peak in the temperature range of 290 °C to 550 °C. This is attributed to the combustion of the activated carbon nanorods. The initial decomposition temperature for the fresh and spent catalysts was similar; however, the final decomposition temperature for the spent catalyst was significantly higher >550 °C. Furthermore, the weight loss for the spent catalyst was 3.7 wt% higher than that of the fresh catalyst, which was due to the oxidation of coke in air (Fig. S3C†). The coke is categorized as hard coke, which decomposed completely at a temperature >550 °C.^61^ The deposited coke will accumulate on the catalyst surface, which covers the active sites on the catalyst and reduces the catalytic DO activity. The negligible loss in DO activity throughout eight reaction runs is an indication that coke formation had an insignificant effect on the active sites of the Co_3_O_4(20%)_–La_2_O_3(20%)_/AC_nano_ catalyst, which confirms that the La_2_O_3_ and Co_3_O_4_ supported AC catalyst exhibits high stability. Notably, the use of carbon as a support in the DO reaction has been widely explored due to its excellent thermal stability. For instance, the use of a bimetallic doped carbon catalyst in the DO of non-edible oil including jatropha oil and WCO was recently explored by our group.^5,6,11^ Ni–Ag/AC, CaO–La_2_O_3_/AC, and Ag_2_O_3_–La_2_O_3_/AC_nano_ were found to be effective in removing the oxygenated compound *via* deCOx pathways and yielded hydrocarbon in the range of 72–89% with product selectively toward *n*-C_15_ + C_17_ fractions (82–93%) and could be reused four to six times (Table 5). Notably, Co_3_O_4(20%)_–La_2_O_3(20%)_/AC_nano_ exhibited excellent catalyst activity with hydrocarbon yield and selectivity of 96% and 93%, respectively. Moreover, the Co_3_O_4(20%)_–La_2_O_3(20%)_/AC_nano_ catalyst showed high catalytic stability with eight times successive reusability with a hydrocarbon yield of >80% and *n*-C_15_ + C_17_ selectivity of >83%. Although the coke formed on the surface of Co_3_O_4(20%)_–La_2_O_3(20%)_/AC_nano_ was more pronounced than that in previous studies, it still exhibited high stability, reusability and catalytic activity. This suggests by the presence of high acidic active sites with a high surface area and pore volume,^12^ which are favorable for the DO reaction.

**Table 5:** Comparison study on catalytic DO with various feeds

No.	Catalyst	Support	Reaction	Feed	FFA (%)	Reaction condition	H/C (yield%)	Selectivity (%)	Reusability	Coke (wt%)	Reference
1	Ni–Ag/AC_CFR_	Coconut fibre residue	DO	JCO	15.4	Catalyst loading: 5 wt%	80	83 (*n*-C_15_ + C_17_)	5	2.5	11
						Temperature: 350 °C					
						Time: 1 h under N_2_ flow					
2	CaO–La_2_O_3_/AC	Walnut shell	DO	WCO	18.4	Catalyst loading: 3 wt%	72	82 (*n*-C_15_ + C_17_)	6	2	5
						Temperature: 330 °C,					
						Time: 3 h under N_2_ flow					
3	Ag_2_O_3_–La_2_O_3_/AC_nano_	Walnut shell	DO	WCO	18.4	Catalyst loading: 1 wt%	89	93 (*n*-C_15_ + C_17_)	6	1.3	6
						Temperature: 350 °C					
						Time: 2 h under N_2_ flow					
4	Co_3_O_4(20%)_–La_2_O_3(20%)_/AC_nano_	Walnut shell	DO	WCO	38.4	Catalyst loading: 1 wt%	96	93 (*n*-C_15_ + C_17_)	8	3.7	Present study
						Temperature: 330 °C					
						Time: 1 h and 5% of water content					

## 4. Conclusion

In the present work, an effective DO reaction was developed by converting triglyceride-based feeds to diesel-like fuel over the Co_3_O_4(*x*)_–La_2_O_3(*y*)_/AC_nano_ catalyst. The improvement in the DO of WCO over the acid–base Co_3_O_4(*x*)_–La_2_O_3(*y*)_/AC_nano_ catalyst was due to the neutralization of the strong acidic sites. DO using a micro-reactor closed system showed better reactivity compared to semi-batch inert flow conditions due to the advantages of high pressure, which is beneficial in maintaining high catalytic activity. The cracking pathway was found to be enhanced with an increase in Co content due to the richness of the high-strength basic and acid sites of the Co_3_O_4(*x*)_–La_2_O_3(*y*)_/AC_nano_ catalyst. Among the Co contents (5–25 wt%), the optimum content of Co for deCOx activity is 20 wt%. The effects of reaction time, catalyst loading, temperature, water and FFA content were further investigated with the optimum DO condition (hydrocarbon fraction = 96%, *n*-C_15_ + *n*-C_17_ = 93%) achieved using 1 wt% catalyst loading within 60 min at 330 °C, 38.4 wt% FFA and 5% water content in the micro-batch closed system. The efficiency of the Co_3_O_4(20%)_–La_2_O_3(20%)_/AC_nano_ catalyst was proven to be capable of deoxygenizing low quality feedstocks with a high FFA (38%) and high water content (5 wt%) under the optimum conditions. The Co_3_O_4(20%)_–La_2_O_3(20%)_/AC_nano_ catalyst showed high stability and reusability up to eight times with the yield and selectivity maintained at >72% and >80% for *n*-C_15_ + C_17_.

## Conflicts of interest

There are no conflicts to declare.

## Supplementary Material

RA-010-C9RA09516K-s001
