# Supplementary material for: Efficient deoxygenation of waste cooking oil over Co3O4–La2O3-doped activated carbon for the production of diesel-like fuel
Source: RSC Adv. 2020 Jan 30;10(9):4996–5009. doi: 10.1039/c9ra09516k (PMC9049065; doi:10.1039/c9ra09516k)
Supplement: RA-010-C9RA09516K-s001 [file RA-010-C9RA09516K-s001.pdf]

Electronic Supplementary Material (ESI) for RSC Advances.
This journal is © The Royal Society of Chemistry 2020

Supplementary Data

List of Figure

1- XPS Spectra of (A) Wide Scan Spectra, (B) O 1s, (C) C 1s,(D) Co2P,(E) P 2p and (F) La 3d5 Core Levels for the $Co_3O_{4(20)}$–$La_2O_{3(20)}$/AC_{nano} catalyst.

2- (A-B) Effect of water content on DO of WCO and (C-D) Effect of FFA of DO of WCO under optimum condition 1wt.%, at 330°C for 60 min under microstructured batch close system.

3- (A) Reusability of $Co_3O_{4(20\%)}$-$La_2O_{3(20\%)}$/AC_{nano} catalyst in DO of WCO at temperature of 330 °C, catalyst amount of 1 wt.% and reaction time of 60 min using microstructured batch close system, (B) XRD diffraction profile (B) TGA profiles for fresh and spent $Co_3O_{4(20\%)}$-$La_2O_{3(20\%)}$/AC_{nano} catalyst after 8th runs.

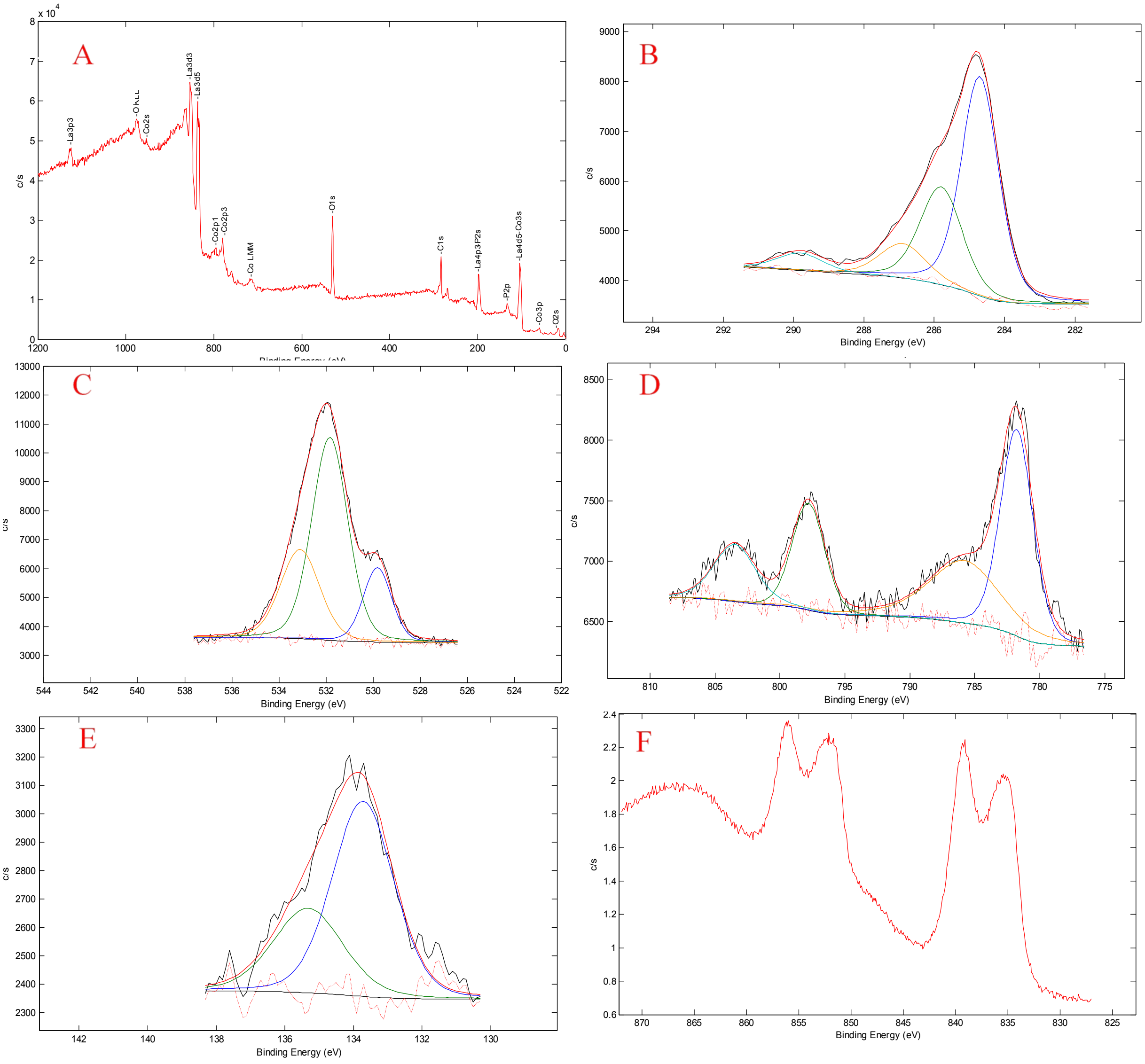

Fig. 1S. XPS Spectra of (A) Wide Scan Spectra, (B) O 1s, (C) C 1s,(D) Co2P,(E) P 2p and (F) La 3d5 Core Levels for the $Co_3O_{4(20)}$–$La_2O_{3(20)}$/AC_{nano} catalyst.

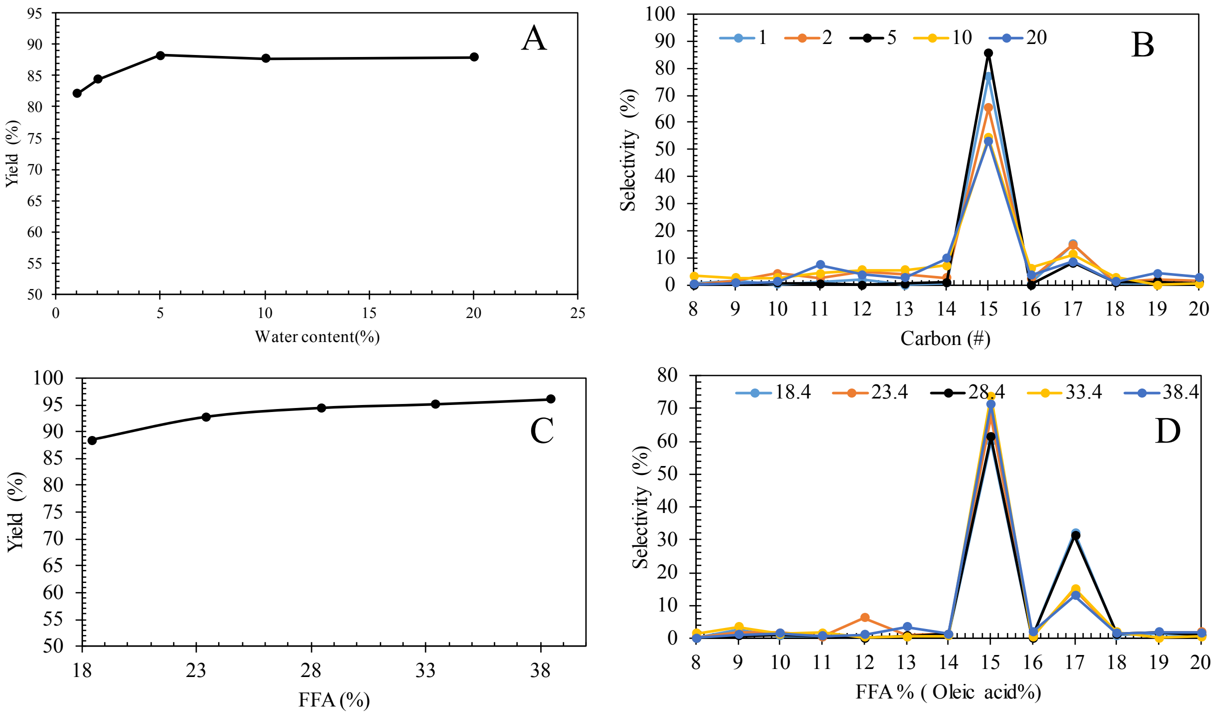

Fig. 2S. (A-B) Effect of water content on DO of WCO and (C-D) Effect of FFA of DO of WCO under optimum condition 1wt.%, at 330°C for 60 min under microstructured batch close system.

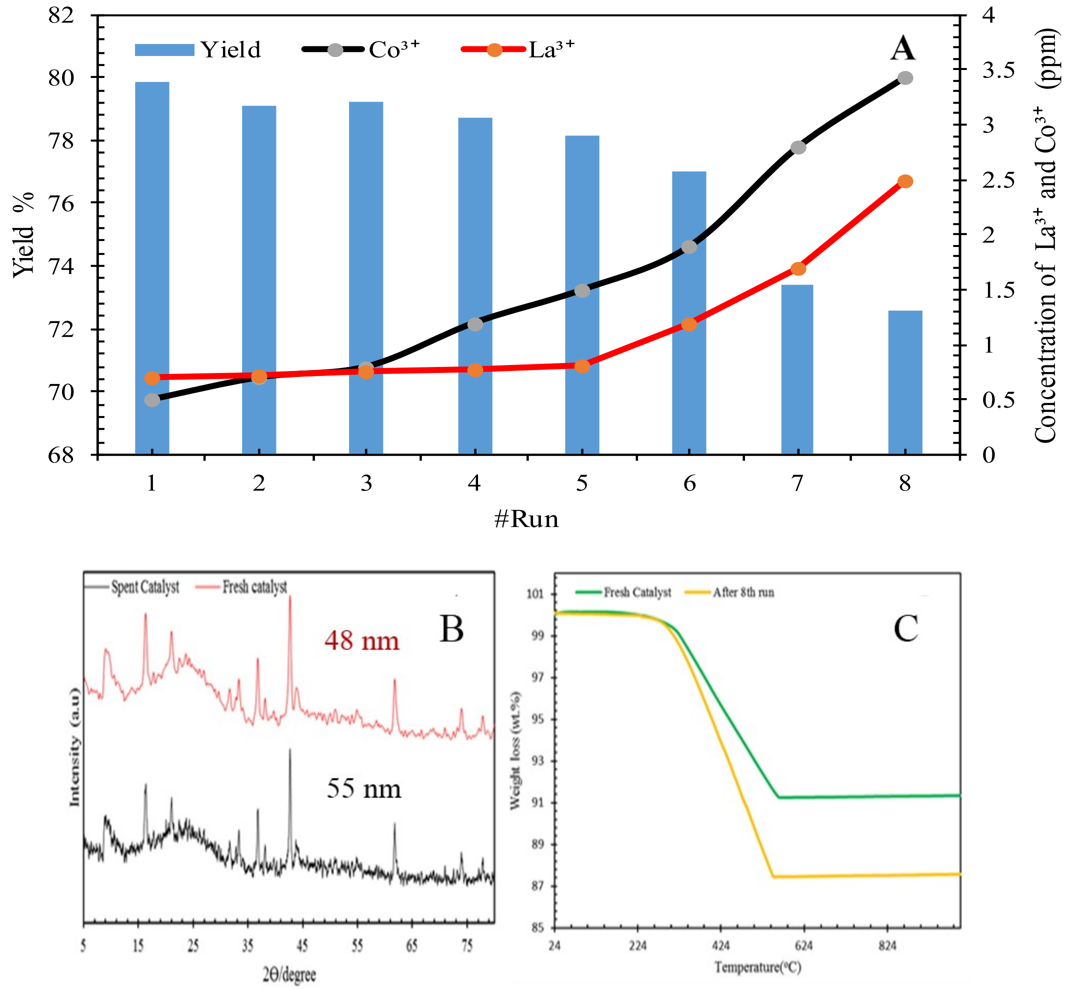

Fig. 3S. (A) Reusability of $Co_3O_{4(20\%)}$-$La_2O_{3(20\%)}$/AC_{nano} catalyst in DO of WCO at temperature of 330 °C, catalyst amount of 1 wt.% and reaction time of 60 min using microstructured batch close system, (B) XRD diffraction profile (C) TGA profiles for fresh and spent $Co_3O_{4(20\%)}$-$La_2O_{3(20\%)}$/AC_{nano} catalyst after 8th runs.

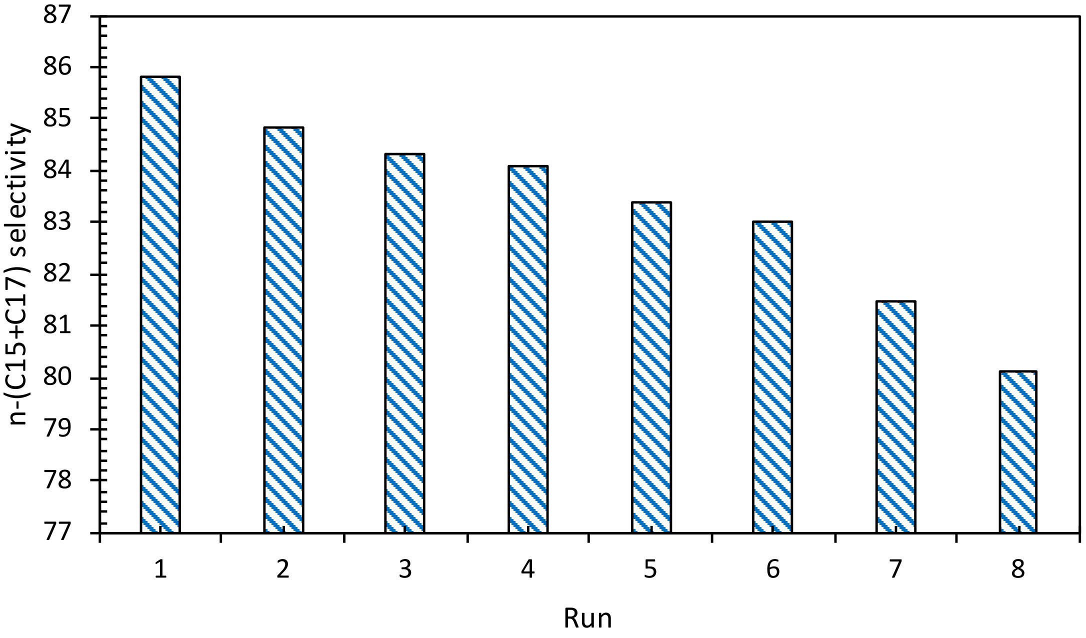

Fig. S4. *n*-(C_{15} +C_{17}) selectivity with the number of DO cycles at at temperature of 330 °C, catalyst amount of 1 wt.% and reaction time of 60 min using microstructured batch close system.
